# A Database-Derived Global Overview of HCV Resistance-Associated Substitutions: Characterizing Genotypic, Regional, and Temporal Heterogeneity

**DOI:** 10.3390/ijms27115068

**Published:** 2026-06-03

**Authors:** Gabriela Tavares Marinho Nunes, Thaís Barbosa Ferreira Sant’Anna, Natalia Motta de Araujo

**Affiliations:** Laboratory of Molecular Virology and Parasitology, Oswaldo Cruz Institute, Oswaldo Cruz Foundation, Rio de Janeiro 21040-900, Brazil; gabrielatmnunes@gmail.com (G.T.M.N.); thaisbfsantanna@gmail.com (T.B.F.S.)

**Keywords:** hepatitis C virus, resistance-associated substitutions, direct-acting antivirals, viral genotypes, database surveillance

## Abstract

Direct-acting antivirals (DAAs) have revolutionized hepatitis C virus (HCV) therapy, yet resistance-associated substitutions (RASs) remain a concern in specific clinical contexts. Here, we present a database-derived global overview of HCV RASs by analyzing 19,449 publicly available sequences across multiple genotypes and subtypes, encompassing both untreated and previously treated infections, in the NS3, NS5A, and NS5B genomic regions. We demonstrate a markedly heterogeneous distribution of RASs shaped by viral genotype, geographic origin, and treatment era. Importantly, RASs against pan-genotypic NS3 protease inhibitors (glecaprevir and voxilaprevir) were rare (generally <1% across genotypes). In contrast, NS5A inhibitors showed greater vulnerability, with the Y93H substitution detected at notable frequencies in major genotypes (3.2–7.0%) and near-universal resistance-associated substitutions (e.g., 100% Q30S) observed in the rare genotype 8. The NS5B nucleotide analogue sofosbuvir retained a high genetic barrier, with the canonical S282T substitution detected only sporadically (2.1% of genotype 4 sequences). At the population level, geographic heterogeneity was evident, with higher RAS frequencies observed in specific regions, alongside pronounced data gaps in high-prevalence areas of Africa. Temporal analyses revealed an increase in NS3 and NS5A RASs following the introduction of first-generation DAAs, with NS5A substitutions persisting into the current interferon-free era, whereas NS5B resistance remained consistently rare across all treatment periods. Together, these findings provide a global, population-level overview of resistance-associated HCV diversity and reinforce the durability of high-barrier regimens while highlighting persistent genotype-specific vulnerabilities with implications for antiviral resistance surveillance and HCV elimination efforts.

## 1. Introduction

Hepatitis C virus (HCV) is an enveloped member of the *Flaviviridae* family with a positive-sense, single-stranded RNA genome of approximately 9.6 kb [1]. This genome encodes a single polyprotein that is co- and post-translationally cleaved into structural and non-structural (NS) proteins. Among the non-structural components, the NS3 protease, the NS5A replication complex, and the NS5B polymerase represent the core machinery of viral replication and the direct targets of contemporary direct-acting antivirals (DAAs) [2,3]. Table 1 summarizes the functional properties and corresponding inhibitor classes for these three key viral targets. Viral output can reach roughly 10^12^ particles per day [4]. Because its RNA-dependent RNA polymerase lacks proofreading activity, this intense replication fuels substantial genetic diversity. As of 2022, eight genotypes and more than 90 subtypes are recognized, with up to 30% nucleotide divergence between genotypes and about 15% between subtypes [5]. Globally, the World Health Organization estimates 50 million people living with chronic HCV infection, nearly one million new infections annually, and 242,000 deaths in 2022, largely due to cirrhosis and hepatocellular carcinoma [6].

DAAs targeting the NS3 protease, the NS5A replication complex, and the NS5B polymerase have transformed HCV therapy, with all-oral, interferon-free regimens achieving sustained virologic response (SVR) rates >95% across diverse clinical contexts [7,8]. Although naturally occurring and treatment-selected resistance-associated substitutions (RASs) can affect antiviral activity in selected scenarios, current evidence and guidelines indicate that baseline resistance testing is generally unnecessary for first-line pan-genotypic combinations such as sofosbuvir/velpatasvir (SOF/VEL) and glecaprevir/pibrentasvir (GLE/PIB), with consideration reserved for specific contexts (e.g., genotype 3 with cirrhosis and Y93H, and rare subtypes). For retreatment after DAA failure, SOF/VEL/voxilaprevir (VOX) is preferred and achieves high SVR across RAS profiles; alternatives such as GLE/PIB plus SOF with or without ribavirin (RBV) may be considered where SOF/VEL/VOX is unavailable [3,9,10]. From a virological standpoint, resistance dynamics differ across drug classes: NS5A inhibitors have a low mutational barrier and selected substitutions can persist for prolonged periods; NS3 protease inhibitor RASs often carry higher fitness costs and tend to wane without drug pressure; and the NS5B nucleotide analogue SOF has a high barrier to resistance, with the canonical S282T being rare and fitness-compromising [3,11].

Against this backdrop, wide regional variation in genotype and subtype distribution, together with inequities in access to testing and treatment, means that the distribution of resistance-associated substitutions can meaningfully influence regimen selection, retreatment strategies, and even procurement policies, particularly in settings with limited therapeutic options or constrained retreatment pathways. While clinical trials and real-world cohorts establish regimen performance, large-scale comparative genomics uniquely quantifies how often clinically relevant substitutions occur in routine populations and how their temporal and geographic patterns evolve, addressing gaps left by prior maps focused on single genotypes or restricted regions.

Given this context, we analyzed a large set of publicly available HCV sequences spanning multiple genotypes and subtypes, encompassing both untreated and previously treated infections, to map RASs across NS3, NS5A, and NS5B. Rather than estimating baseline resistance prevalence in treatment-naïve populations, this approach captures the resistance-relevant viral diversity represented in international public databases, reflecting a composite of natural polymorphism, historical treatment exposure, and global sequencing practices. By quantifying site-specific frequencies across genotypes/subtypes, estimating target-specific resistance burdens, and comparing patterns across regions and treatment eras, we provide a descriptive population-level, database-derived global overview of RAS distribution that may help inform treatment choices and surveillance priorities.

## 2. Results

**Figure 1:**
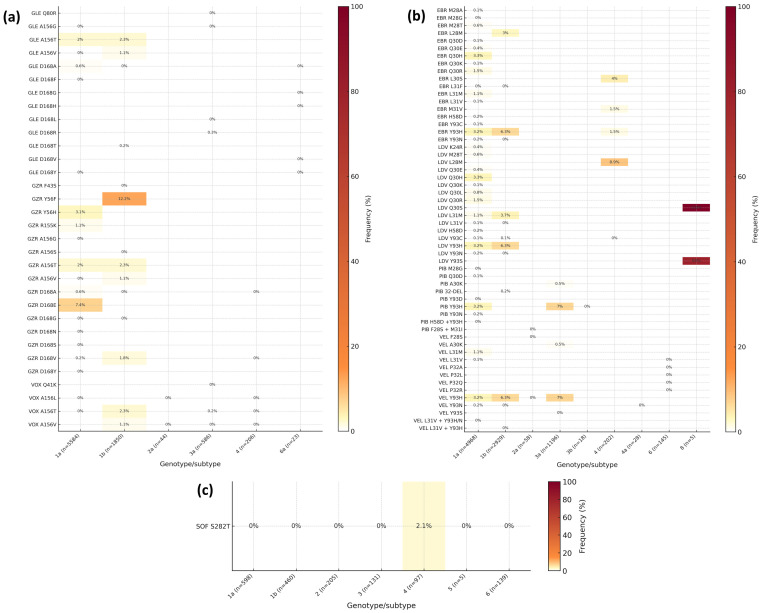
Frequency of resistance-associated substitutions (RASs) in hepatitis C virus (HCV) across three genomic regions: (**a**) NS3, including Glecaprevir (GLE), Grazoprevir (GZR), and Voxilaprevir (VOX); (**b**) NS5A, including Elbasvir (EBR), Ledipasvir (LDV), Pibrentasvir (PIB), and Velpatasvir (VEL); and (**c**) NS5B, represented by Sofosbuvir (SOF). Heatmaps display the proportion of sequences harboring each RAS by HCV genotype/subtype, expressed as percentages. Blank cells indicate RASs not associated with resistance in the given genotype/subtype. Numbers in parentheses below each column indicate the sequences analyzed per genotype/subtype.

Our analysis of a global dataset of 19,449 HCV sequences provided a detailed map of RASs, which encompasses both natural polymorphisms and potential treatment-selected mutations, across the major targets of DAAs. The frequencies of key RASs in the NS3 protease, NS5A replication complex, and NS5B polymerase are summarized in Figure 1.

The prevalence of RASs against the pan-genotypic NS3 protease inhibitors GLE and VOX was generally low (<1%) across all major genotypes (Figure 1a). For GLE, the most frequently observed substitutions were A156T and A156V in genotypes 1a and 1b, though still at low frequencies (1.1–2.3%). In stark contrast, analysis for GZR, a first-generation protease inhibitor, revealed high-prevalence polymorphisms in specific genotypes. Notably, the Y56F substitution was present in 12.2% of genotype 1b sequences, and the D168E substitution was found in 7.4% of genotype 1a sequences. For the salvage regimen VOX, RASs remained uncommon, with low-frequency occurrences primarily in genotype 1b. The A156T and A156V substitutions were observed in 2.3% and 1.1% of 1b sequences, respectively. In contrast, these and other VOX-associated RASs were virtually absent in all other genotypes, with only a single A156T substitution detected in one genotype 3a sequence (0.2%). The NS5A region exhibited a markedly different and more concerning resistance profile, with several RASs present at clinically significant frequencies across multiple genotypes and inhibitors (Figure 1b). The Y93H substitution was a major hotspot, observed in 3.2% of 1a, 6.3% of 1b, and 7% of 3a sequences, conferring resistance to EBR, LDV, PIB, and VEL. In genotype 1a, the Q30H substitution was also prevalent at 3.3%. A striking finding was the very high prevalence of certain RASs in less common genotypes; for instance, the Q30S and Y93S substitutions were present in 100% and 80% of the few available genotype 8 sequences, respectively, for LDV. Furthermore, while the second-generation NS5A inhibitors PIB and VEL showed a broader genetic barrier, the persistence of Y93H in genotypes 1 and 3 at notable frequencies underscores a potential vulnerability. Analysis of the NS5B polymerase region, targeted by the nucleotide inhibitor SOF, confirmed its high genetic barrier to resistance (Figure 1c). The canonical RAS S282T was exceptionally rare, detected in only 2.1% of genotype 4 sequences and entirely absent (0%) in all other major genotypes, including 1a, 1b, 2, 3, 5, and 6.

**Figure 2:**
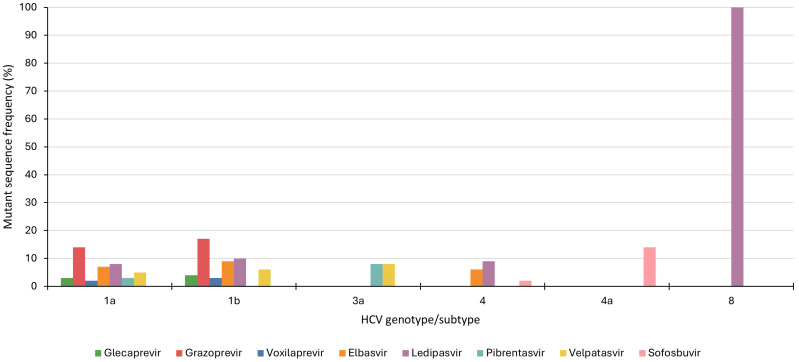
Frequency of hepatitis C virus (HCV) sequences harboring at least one resistance-associated substitution (RAS) across genotypes/subtypes. Color-coded bars represent direct-acting antivirals (DAAs) targeting the genomic regions NS3, NS5A, and NS5B.

Figure 2 illustrates the composite frequency of sequences harboring at least one RAS across major HCV genotypes, revealing profound genotypic disparities in the population-level RAS landscape. This analysis shows that genotypes 1a and 1b carry the highest aggregate burden of RASs across multiple drug classes. This is particularly evident for the NS3 inhibitor GZR (14% and 17%, respectively) and several NS5A inhibitors. In contrast, genotype 3a displays a distinct signature, with a notable 8% RAS frequency for the pan-genotypic NS5A inhibitors PIB and VEL, while showing negligible RASs for NS3 inhibitors. The data also highlight the unique profiles of less common genotypes; genotype 4a shows a singularly high RAS frequency (14%) for SOF, and genotype 8 demonstrates an extreme vulnerability, with 100% of sequences carrying a RAS against LDV. This comparative genomic overview underscores that the population-level RAS reservoir is intrinsically shaped by viral genotype, with genotypes 1a/1b, 3a, 4a, and 8 presenting distinct and significant challenges to specific DAA classes.

**Figure 3:**
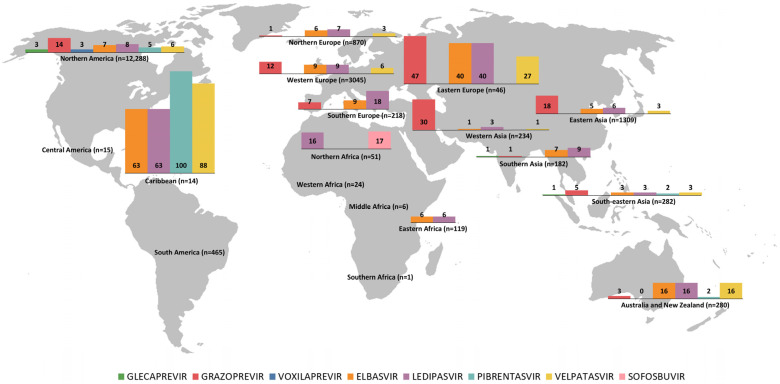
Geographic distribution of hepatitis C virus (HCV) sequences harboring at least one resistance-associated substitution (RAS) for the DAAs analyzed in this study, based on 19,449 sequences from the Los Alamos database. Numbers at the top of each column correspond to the percentage of mutant sequences, while numbers in parentheses indicate the total sequences analyzed in each geographic region. Regions without bars represent cases where no mutant sequences were detected for any DAA. The map was reconstructed using a base image from Wikimedia Commons https://commons.wikimedia.org/wiki/File:BlankMap-World-noborders.png (accessed on 15 March 2025); the map is similar but not identical to the original and is used here for illustrative purposes only.

Figure 3 reveals a highly heterogeneous geographic distribution of HCV sequences harboring at least one RAS, with several regions emerging as significant hotspots for specific drug classes. A remarkable finding is the extremely high RAS frequencies in the Caribbean and Eastern Europe, albeit based on limited sequence counts (*n* = 14 and *n* = 46, respectively). In the Caribbean, the analyzed sequences exhibited notably high RAS frequencies against NS5A inhibitors, with 63% for EBR and LDV, 88% for VEL, and all sequences (100%) harboring a RAS for PIB. Similarly, Eastern Europe showed high prevalence for EBR/LDV at 40%. For the NS3 protease inhibitors, Eastern Asia (18%), Western Asia (30%) and Eastern Europe (47%) stood out as regions with notably high prevalence of RASs against GZR. In contrast, RASs for the newer pan-genotypic protease inhibitors GLE and VOX remained below 3% in all regions, confirming their robust profile. A notable exception was Northern Africa, which reported a 17% RAS frequency for SOF, a unique finding not observed elsewhere. North America and Western Europe, which collectively contributed the largest number of sequences (*n* = 12,288 and *n* = 3045, respectively), showed moderate, broad-spectrum RAS profiles, with GZR (14% and 12%, respectively) and various NS5A inhibitors (5–9%) being most affected. An unexpected observation was noted in South America (*n* = 465), where no resistance-associated substitutions were detected across any DAA class, despite the relatively large number of sequences available. By contrast, the absence of detected RASs in regions with very limited sequence representation, such as Central America (*n* = 15), Western Africa (*n* = 24), Middle Africa (*n* = 6), and Southern Africa (*n* = 1), is more plausibly explained by critical undersampling, precluding meaningful regional inferences.

**Figure 4:**
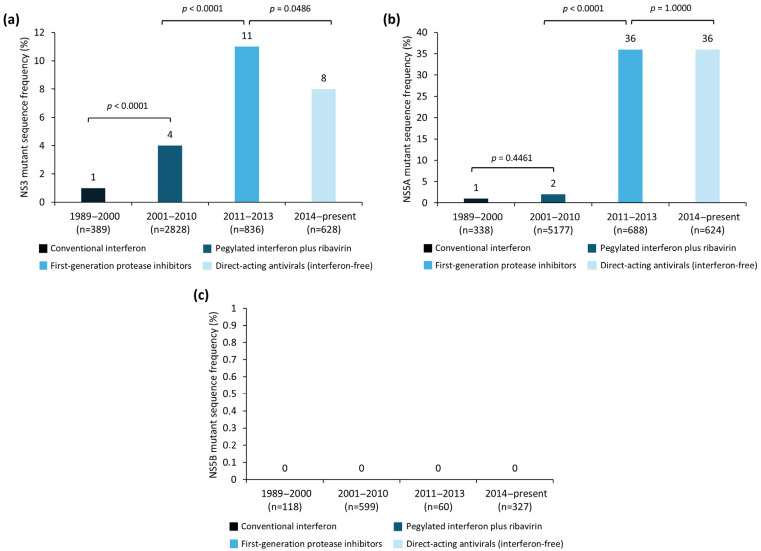
Frequency of hepatitis C virus (HCV) sequences harboring at least one resistance-associated substitution (RAS) in (**a**) NS3, (**b**) NS5A, and (**c**) NS5B genomic regions over time, stratified by treatment era. Significant differences between treatment eras are indicated by *p*-values. Color-coded bars represent treatment regimens: conventional interferon (1989–2000), pegylated interferon plus ribavirin (2001–2010), first-generation protease inhibitors (2011–2013), and interferon-free direct-acting antiviral (DAA) regimens (2014–present).

Figure 4 presents the temporal distribution of HCV sequences harboring at least one RAS across the NS3, NS5A, and NS5B genomic regions, stratified by four major treatment eras. To evaluate this, we analyzed a total of 12,612 sequences that contained information on the year of sample collection. The sequences were grouped into time periods corresponding to distinct treatment eras: 1989–2000 (conventional interferon therapy), 2001–2010 (pegylated interferon plus ribavirin), 2011–2013 (first-generation protease inhibitors), and 2014–present (interferon-free DAA regimens). In the NS3 region (Figure 4a), the frequency of RASs remained low during the interferon-based eras (1989–2010), as interferon-based therapies do not impose direct, target-specific selective pressure on the NS3 protease. A marked increase was observed following the introduction of first-generation protease inhibitors (2011–2013), with RAS frequencies rising significantly (*p* < 0.05). In the interferon-free DAA era (2014–present), the frequency of NS3 RASs declined slightly but stabilized, likely due to the adoption of pan-genotypic regimens with higher resistance barriers, such as GLE and VOX, which replaced earlier protease inhibitors. For NS5A (Figure 4b), a significant rise in RAS frequency was already apparent during the first-generation protease inhibitor era (2011–2013), indicating an early emergence of these substitutions prior to the widespread use of interferon-free NS5A-containing regimens. This elevated frequency has persisted stably into the current DAA era (2014–present). In the NS5B region (Figure 4c), RAS frequencies remained consistently minimal across all treatment eras, with no changes over time.

## 3. Discussion

This study drew upon a large, globally sourced repository to chart the distribution of RASs in HCV NS3, NS5A, and NS5B across genotypes/subtypes, geographic regions, and treatment eras. A key interpretive consideration is that the dataset includes sequences from both treatment-naïve and treatment-experienced individuals; therefore, the frequencies reported here reflect the population-level ecology of resistance-relevant diversity captured in public databases, rather than true baseline prevalence. This mixed clinical background aligns with established virological dynamics, in which NS5A substitutions often persist for prolonged periods after NS5A-containing regimen failure, whereas many NS3 and NS5B substitutions tend to revert once drug pressure is removed [12,13]. As a result, database-level snapshots likely represent a composite of natural polymorphism, historical drug exposure, and substitution-specific fitness costs, rather than a single underlying clinical state. Our findings confirm the high genetic barrier to resistance of modern pan-genotypic regimens such as GLE/PIB and SOF/VEL but reveal a complex and heterogeneous landscape of resistance-relevant diversity shaped by viral genotype, geography, and treatment history. These population-level insights have direct implications for treatment policy, surveillance, and retreatment strategies.

The low prevalence of RASs against the pan-genotypic NS3 protease inhibitors GLE and VOX (<1% across most genotypes) is a reassuring confirmation of their high genetic barrier. It confirms that the viral variants capable of resisting these newer agents are rare in the broader population, which supports their robust clinical performance and under-pins their recommendation as first-line and salvage therapies, respectively [9,10]. In stark contrast, the high prevalence of polymorphisms like Y56F in genotype 1b (12.2%) and D168E in 1a (7.4%) for the first-generation inhibitor GZR underscores how resistance-relevant viral diversity can compromise the efficacy of earlier agents and the success of subsequent drug design in overcoming these common variants. Consistent with this, Schnell et al. (2018) found Y56F in over 30% of 1b sequences worldwide, with frequencies exceeding 50% in some European cohorts [14].

The most critical insights emerge from the NS5A region, consistently identified as the Achilles’ heel of DAA therapy due to the low barrier to resistance and long persistence of its RASs [3]. Our observation that Y93H is present at notable frequencies in major genotypes (1a, 1b, and 3a) is particularly significant. Although second-generation NS5A inhibitors like PIB and VEL have an improved resistance profile, the presence of Y93H in 7% of genotype 3a sequences is non-trivial, especially given that this genotype is often associated with faster fibrosis progression and historically lower SVR rates with some regimens [15,16]. This finding corroborates and quantifies on a global scale the warnings from earlier studies about the impact of this substitution in genotype 3 [17]. Furthermore, the extreme RAS prevalence in rarer genotypes, such as the universal presence of Q30S in the available genotype 8 sequences for LDV, exposes a critical gap in the “pan-genotypic” paradigm. While current pan-genotypic regimens are highly effective, our data suggest that intrinsic resistance in understudied genotypes could lead to unexpected virological failures, a point less emphasized in the broader literature but crucial for global elimination efforts.

For NS5B, our findings corroborate the scarcity of substitutions with meaningful impact on the nucleotide analogue backbone used in contemporary combinations. The canonical SOF-associated S282T remains rare at baseline and infrequent even at failure across clinical trial programs; when it does emerge under suboptimal pressure, it confers resistance but carries a substantial fitness cost and often reverts after treatment cessation, which helps explain the very low NS5B signal in our mixed dataset comprising sequences from both treatment-naïve and treatment-experienced individuals. These features are well established in resistance and clinical studies, including the sofosbuvir phase 2/3 program and dedicated analyses of S282T selection and reversion [18,19]. Together with the high barrier to resistance of nucleotide analogues and the excellent real-world performance of pan-genotypic SOF-based regimens, this evidence supports the limited role for routine NS5B resistance testing outside specific failure analyses, as reflected in contemporary AASLD–IDSA guidance [9].

Our comparative analysis of RASs across HCV genotypes/subtypes revealed pronounced genotype-specific disparities with clear therapeutic implications. Genotype 8 exhibited the highest overall prevalence of RASs, with universal (100%) Q30S and high (80%) Y93S among NS5A substitutions, findings consistent with reduced LDV susceptibility at these positions; however, clinical efficacy data in GT8 remain limited and should be interpreted cautiously [20,21,22]. Genotype 1b also showed elevated RAS frequencies, including 17% to GZR, driven predominantly by NS3-Y56F (12.2%), a substitution documented to reduce GZR susceptibility in this genotype [23,24], and 9–10% to EBR/LDV. In our dataset, RAS frequencies in genotype 1a were broadly similar to 1b (e.g., GZR 14% vs. 17%; LDV 8% vs. 10%), supporting that both subtypes carry potentially relevant NS3/NS5A polymorphisms. Genotype 3a presented an 8% frequency of RASs to PIB and VEL, reflecting the presence of A30K and Y93H polymorphisms that reduce NS5A inhibitor activity and are enriched after DAA exposure [20,25]. While limited by small sample sizes in some subtypes, these genotype-specific patterns underscore the heterogeneous distribution of RASs and reinforce the rationale for prioritizing high-barrier pan-genotypic regimens, such as GLE/PIB or SOF/VEL/VOX, in settings lacking routine genotyping or resistance testing.

Our geographic analysis reveals a heterogeneous global distribution of RASs to DAAs. This heterogeneity likely reflects a composite of factors, including the underlying genotype/subtype lineage structure of HCV, uneven sequencing density across regions, and divergent local treatment histories and access to DAAs. The notably high apparent RAS frequencies in settings such as the Caribbean and Eastern Europe, albeit based on limited sample sizes, suggest the possibility of localized epidemics with distinct resistance profiles that could undermine the performance of specific DAA classes in those populations. This concern is consistent with work showing that regional subtype structure and founder effects can shape local resistance landscapes [14,26]. In contrast, regions with extensive sequencing efforts, including North America and Western Europe (*n* = 12,288 and *n* = 3045, respectively), displayed moderate, broad-spectrum RAS patterns, plausibly reflecting longer and more heterogeneous histories of DAA uptake in these settings. A particularly notable signal in our dataset is the absence of detected RASs in the well-sampled South American cohort (*n* = 465). While this finding could suggest a comparatively susceptible viral landscape in publicly available sequences, it should be interpreted with caution, as multiple studies have reported baseline RASs at low-to-moderate frequencies in South America, particularly at NS5A positions (e.g., A30K and Y93H) in Brazil, Colombia, and Argentina, and occasionally in NS3 among Brazilian genotype 1–infected patients [27,28,29,30,31,32]. To better understand this unexpected result, we performed a closer inspection of this regional dataset and found that 435 of the 465 sequences corresponded to intra-host clones derived from only eight individuals (four non-responders and four end-of-treatment responders) from a single study [33]. This extensive clonal overrepresentation resulted in a disproportionate contribution of a limited number of viral populations, effectively masking the presence of resistance-associated substitutions at the regional level. Importantly, real-world studies from the region consistently report high sustained virologic response rates (>90–95%) with current DAA regimens, supporting the notion that baseline RASs do circulate in South America but have not generally compromised the effectiveness of pan-genotypic strategies, except in specific high-risk contexts such as genotype 3 infection with baseline NS5A RASs [34,35]. In contrast, the absence of detected RASs in regions with very limited sequence representation, such as Central America (*n* = 15), Western Africa (*n* = 24), Middle Africa (*n* = 6), and Southern Africa (*n* = 1), must not be misinterpreted as a true absence of resistance. Instead, this is more plausibly explained by critical regional undersampling and severe data voids, which preclude meaningful regional inferences. Strengthening genomic surveillance in these specific underrepresented, high-burden areas remains a blind spot that must be prioritized.

The temporal analysis of RAS prevalence illustrates how successive treatment eras shaped the viral resistance landscape. In our dataset, RASs were rare during the interferon eras (conventional IFN and pegylated IFN + ribavirin) and increased substantially only with the introduction of first-generation protease inhibitors, while NS5B remained essentially devoid of detectable resistance substitutions across all eras (Figure 4). This pattern aligns with the pre-DAA literature showing that IFN-based regimens exerted broad, non-specific antiviral pressure without selecting class-specific escape pathways, so naturally occurring polymorphisms rarely reached appreciable prevalence or clinical salience [36]. The sharp rise in RAS frequency during the first-generation protease inhibitor era can be understood even though telaprevir- and boceprevir-specific sites were not enumerated here: (i) early NS3 inhibitors had a low genetic barrier, rapidly selecting canonical substitutions (e.g., R155, A156, D168) that also confer varying degrees of cross-resistance to later PIs, thereby inflating the overall NS3-RAS signal; (ii) deep sequencing and programmatic resistance testing expanded during 2011–2013, improving detection; and (iii) a transient shift toward genotype 1-dominant treatment cohorts, historically more exposed to PIs, increased the sampling of selected variants [37,38,39]. From the interferon-free DAA era onward (2014–present), the decline in NS3 RAS prevalence (Figure 4a) aligns with replacement of early PI-anchored strategies by pan-genotypic regimens (e.g., GLE/PIB; SOF/VEL/VOX) that sustain high cure rates and exhibit higher resistance barriers at the regimen level, reducing the opportunity for persistent NS3 escape. Guidance documents and clinical experience over this period emphasize the diminishing role of baseline PI RASs in most retreatment decisions outside specific scenarios [9]. In contrast, NS5A shows an early surge followed by a persistent high plateau (Figure 4b). This pattern may not reflect direct drug selection during the first-generation PI era but instead could stem from the expansion and long-term persistence of naturally occurring NS5A substitutions (e.g., L31M/V, Y93H, Q30R/H, A30K) that pre-dated the introduction of NS5A inhibitors. The frequent virologic failures observed with the low-barrier telaprevir/boceprevir regimens might have provided prolonged windows of within-host replication in which such variants could rise to detectable frequencies, after which their relatively low fitness cost may have enabled long-term maintenance even in the absence of NS5A-directed pressure. Under this interpretation, the apparent early “emergence” of NS5A RASs reflects a reshaping of existing diversity, rather than class-specific selection, helping explain the stable high plateau observed in later interferon-free eras. Such long-lived NS5A substitutions have been documented beyond 96 weeks after failure and can complicate retreatment choices, a fact embedded in modern guidance (e.g., genotype 3 with baseline Y93H) [13,40]. By comparison, the NS5B polymerase profile (Figure 4c) remains flat and negligible across eras, consistent with the exceptionally high genetic barrier of SOF [18,20].

Several limitations of this study should be acknowledged. First, our analysis relied on publicly available HCV sequences, which may not fully represent global viral diversity due to uneven sequencing efforts, regional deposition biases, and heterogeneous study designs. Second, public databases incompletely report clinical metadata, including treatment history, precluding systematic stratification of sequences from treatment-naïve and treatment-experienced individuals. As a result, the frequencies reported here do not represent baseline resistance prevalence but rather reflect population-level resistance-relevant diversity shaped by a composite of natural polymorphism and historical treatment exposure. Third, large sequence repositories may disproportionately include multiple intra-host variants from a limited number of individuals, as illustrated by the South American dataset, in which the majority of sequences corresponded to intra-host clones derived from few patients. Because standardized patient-level identifiers are generally unavailable, systematic global de-duplication (i.e., the consistent identification and collapsing of multiple intra-host sequences derived from the same individual across the dataset) is not feasible. Nevertheless, the extensive dataset examined in this study likely reduces the impact of these limitations to some degree. Furthermore, since public databases primarily consist of fragment-specific sequences (NS3, NS5A, or NS5B), tracking linked cross-class resistance within the same viral population remains challenging. While intra-target combinations were evaluated (e.g., NS5A double mutants), the lower frequency of complete genomes relative to partial sequences limits large-scale, population-level estimates of multiclass resistance.

In summary, this large-scale comparative analysis shows that resistance-relevant diversity in HCV is strongly shaped by genotype lineage, geographic distribution, and historical treatment exposure, rather than being uniformly rare or clinically negligible across populations. Genotype 1a/1b, 3a, 4a and 8 displayed distinctive resistance signatures across NS3, NS5A and NS5B, underscoring that the impact of RASs is context-dependent and varies by drug class and viral background. At the same time, the consistently low burden of substitutions against high-barrier regimens, particularly GLE/PIB and SOF/VEL-based combinations, reinforces their robustness as first-line and retreatment options. These findings support current guideline recommendations that reserve baseline resistance testing for selected high-risk situations rather than routine use. However, the marked geographic and genotype-specific heterogeneity we observed, together with substantial sequencing gaps in many high-burden regions, indicates that strengthened genomic surveillance remains essential to sustaining the effectiveness of pan-genotypic DAA strategies and ensuring equitable progress toward global HCV elimination goals.

## 4. Materials and Methods

### 4.1. Selection of Direct-Acting Antivirals and Resistance-Associated Substitutions

We defined the scope of DAAs according to the most recent guidance from the American Association for the Study of Liver Diseases–Infectious Diseases Society of America (AASLD–IDSA) [9] and the European Association for the Study of the Liver (EASL) [10], restricting the analysis to currently recommended agents: GLE, GZR, VOX, EBR, LDV, PIB, VEL, and SOF. To ensure clinical interpretability, we cross-referenced the AASLD–IDSA resistance guidance [20] with the official prescribing information of widely used fixed-dose combinations, including (Epclusa^®^ [SOF/VEL], Harvoni^®^ [LDV/SOF], Mavyret^®^ [GLE/PIB], Vosevi^®^ [SOF/VEL/VOX], and Zepatier^®^ [EBR/GZR]) when compiling the list of candidate RASs. Inclusion followed a two-step procedure. First, we cataloged substitutions reported in drug labels to reduce susceptibility in replicon assays, noting the genotype/subtype context. Second, we screened substitutions observed among patients with documented DAA failure in clinical studies and retained only those meeting fold-change (FC) thresholds consistent with prior syntheses: FC > 100 for first-generation NS5A inhibitors (LDV) and FC > 10 for second-generation DAAs (GLE, GZR, VOX, EBR, PIB, and VEL), as summarized in prior comprehensive reviews [22,41]. The final NS3 set comprised Q41K, F43S, Y56F/H, Q80R, R155K, A156G/L/S/T/V, and D168A/E/F/G/H/L/N/R/S/T/V/Y. The NS5A set comprised K24R, F28S, L28M, M28A/T/G, A30K, L30S, Q30D/E/H/K/L/R/S, L31F/M/V, M31V, P32A/L/Q/R, the P32 deletion (P32Del), H58D, Y93C/D/H/N/S, and the clinically reported double substitutions F28S+M31I, L31V+Y93H/N, and H58D+Y93H. For the NS5B nucleotide analogue SOF, only S282T was included. Unless otherwise indicated, residue numbering and nomenclature follow the AASLD–IDSA guidance and the corresponding drug labels.

### 4.2. HCV Genomic Sequences

HCV NS3 (*n* = 19,046), NS5A (*n* = 12,557), and NS5B (*n* = 3665) sequences were retrieved from the Los Alamos HCV Sequence Database as a precomputed multiple-sequence alignment in FASTA format (https://hcv.lanl.gov/content/sequence/HCV/ToolsOutline.html, accessed on 22 January 2025). Metadata on geographic origin and sampling date were extracted from the database annotations. We then restricted the dataset to sequences belonging to genotypes/subtypes with reported resistance at the corresponding target region and with country of origin available; the resulting analysis sets comprised a total of 19,449 sequences (*n* = 8293 for NS3, *n* = 9521 for NS5A, and *n* = 1635 for NS5B). Sequences were further grouped into 21 geographic subregions according to the United Nations Statistical Division (UNSD) M49 classification (https://unstats.un.org/unsd/methodology/m49/#geo-regions, accessed on 30 January 2025). For temporal analysis, we retained only sequences with a documented sample collection date, yielding *n* = 4681 for NS3, *n* = 6827 for NS5A, and *n* = 1104 for NS5B.

### 4.3. Mutational and Statistical Analysis

Multiple-sequence alignments were inspected and curated in MEGA11 (Molecular Evolutionary Genetics Analysis, v11) [42]. We then constructed a spreadsheet dataset (Microsoft Excel) containing, for each sequence, the accession number, RAS position and observed amino-acid substitution, country of origin, and specimen collection date (Supplementary Table S1, sheets ‘Mutational-Geographic Analysis’ and ‘Temporal Analysis’). Mutant sequence frequencies were compared using Fisher’s exact test, and *p*-values for multiple comparisons were adjusted using the Benjamini–Hochberg False Discovery Rate (FDR) procedure (R software version 4.4.1, R Foundation for Statistical Computing, Vienna, Austria). A *p*-value < 0.05 was considered statistically significant.

## Figures and Tables

**Table 1:** Functional properties of HCV non-structural proteins and respective direct-acting antiviral (DAA) classes.

Gene Product	Main Biological Function in Viral Life Cycle	Targeted DAA Class (Examples in Study)
NS3 Protease	A serine protease complex responsible for the cleavage of the viral polyprotein junctions; also plays a critical role in impairing host innate immune signaling pathways.	Glecaprevir, Grazoprevir, Voxilaprevir
NS5A Protein	A zinc-binding phosphorylated protein essential for organizing the viral replication complex, regulating RNA replication, and modulating viral assembly and release.	Elbasvir, Ledipasvir, Pibrentasvir, Velpatasvir
NS5B Polymerase	An RNA-dependent RNA polymerase (RdRp) that drives the synthesis of the complementary negative-strand RNA and subsequent genomic positive-strand RNA.	Sofosbuvir

## Data Availability

The original contributions presented in this study are included in the article/Supplementary Materials. Further inquiries can be directed to the corresponding author.

## Supplementary Materials

The following supporting information can be downloaded at: https://www.mdpi.com/article/10.3390/ijms27115068/s1.

## Abbreviations

The following abbreviations are used in this manuscript:

AASLD	American Association for the Study of Liver Diseases
DAAs	Direct-acting antivirals
EASL	European Association for the Study of the Liver
EBR	Elbasvir
GLE	Glecaprevir
GZR	Grazoprevir
HCV	Hepatitis C virus
IDSA	Infectious Diseases Society of America
IFN	Interferon
LDV	Ledipasvir
PIB	Pibrentasvir
RASs	Resistance-associated substitutions
RBV	Ribavirin
SOF	Sofosbuvir
SVR	Sustained virologic response
UNSD	United Nations Statistical Division
VEL	Velpatasvir
VOX	Voxilaprevir
